# Delayed presentation of cannabis induced pancreatitis

**DOI:** 10.1002/ccr3.5595

**Published:** 2022-03-17

**Authors:** David Song, Harinivaas Shanmugavel Geetha, Samkit Jain, Jonathan Vincent Reyes, Vikash Jaiswal, Gaurav Nepal, Joseph Lieber

**Affiliations:** ^1^ Department of Internal Medicine Icahn School of Medicine at Mount Sinai Elmhurst Hospital Center New York New York USA; ^2^ Department of Internal Medicine at Saint Vincent Hospital Worcester Massachusetts USA; ^3^ AMA School of Medicine Makati Philippines; ^4^ Rani Primary Health Care Centre Biratnagar Nepal

**Keywords:** acute inflammation, acute pancreatitis, cannabis

## Abstract

A thorough history and identifying risk factors are pivotal in establishing the cause of pancreatitis and preventing recurrences to curb the incidence of chronic pancreatitis and/or pancreatic cancer.

## Graphical abstract

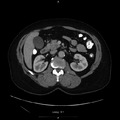

## 1 INTRODUCTION

The definition for acute pancreatitis (AP) was first proposed by the Atlanta classification system in 1992. Subsequently, a more revised classification system to allow for a consistent worldwide classification using definitive imaging criteria was established in 2012. The revised Atlanta classification requires that two or more of the following criteria be met for the diagnosis of acute pancreatitis: (a) abdominal pain suggestive of pancreatitis, (b) serum amylase or lipase level greater than three times the upper normal value, or (c) characteristic imaging findings. Since the implementation of this criteria, the diagnosis of AP has been streamlined and has led to earlier diagnosis and institution of management which is vital during the initial 24–48 hours of manifestation.

The diagnosis and management of AP is pivotal since it is the leading cause of gastrointestinal related hospitalization in the United states.^1^ The incidence of AP continues to increase across the world. It is unclear if this is attributed to a true increase in the number of cases or increased detection of cases of AP. Recently in the United States, the population incidence has been cited as 600–700 per 100,000 people and approximately 200,000–250,000 hospital discharges per year from AP.^2^ Although alcohol and gallstones constitute the major causes of pancreatitis, other causes of pancreatitis are numerous such that it is often difficult to identify a definite etiology of the pancreatitis. However, it is important to establish the etiology of pancreatitis to prevent recurrent episodes that ultimately predispose patients the risk of developing chronic pancreatitis and pancreatic cancer.^3^

## 2 CASE PRESENTATION

The patient is a 64‐year‐old woman with a past medical history of pre‐diabetes, tobacco use disorder, and cannabis use disorder who presented to the emergency department (ED) with 2 weeks of worsening diffuse abdominal pain associated with nausea. She stated that the pain was worse after eating meals, and she took ibuprofen 400 mg once and several doses of bismuth subsalicylate liquid with no relief. The day prior to admission, she reported one episode of non‐biliary, non‐bloody emesis, which prompted her admission to the hospital. She denied any alcohol use, but endorsed a smoking history and cannabis use for the past 20 years. On arrival, she was afebrile, not tachycardic, and saturating well on room air. The abdominal examination revealed a soft, nondistended abdomen with epigastric and left upper quadrant palpable tenderness.

**FIGURE 1:**
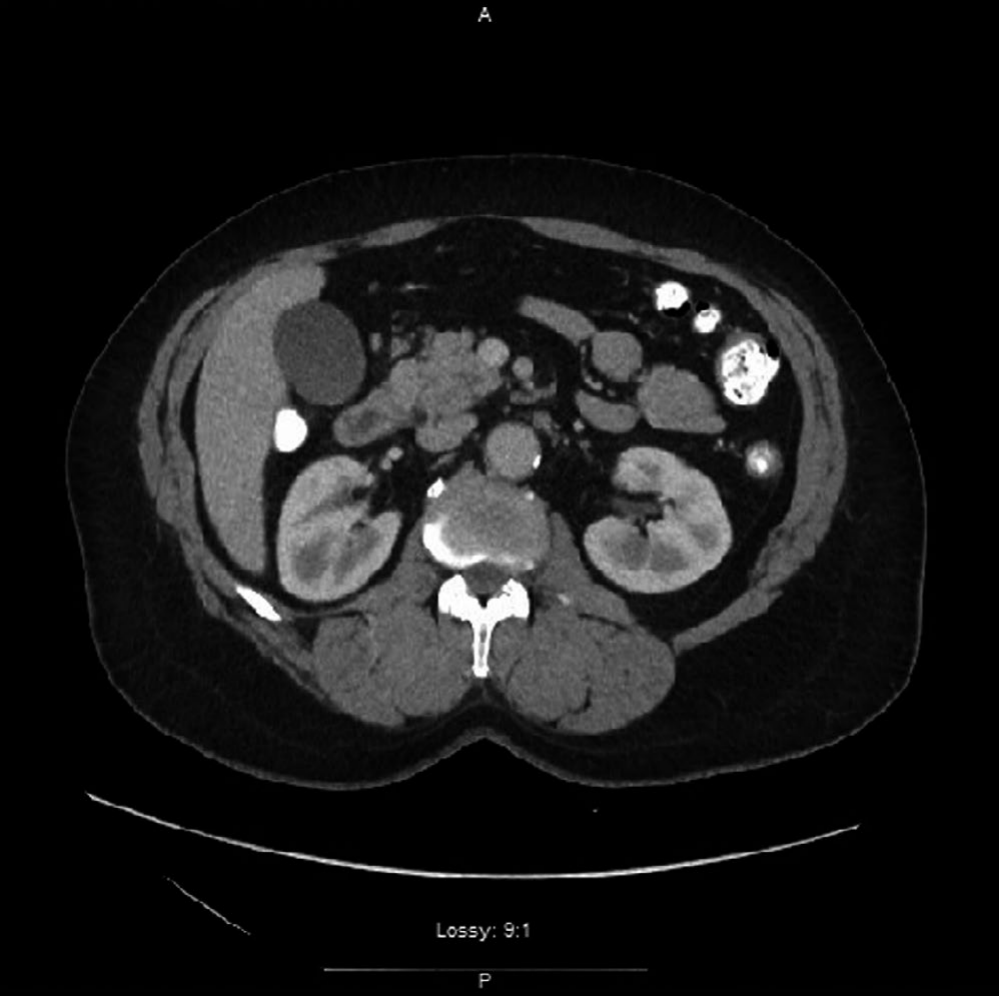
CT Abdomen & Pelvis with contrast demonstrating inflammation surrounding the middle portion of the duodenum and head of the pancreas suggesting acute pancreatitis. (Axial View)

In the ED, the laboratories were notable for an elevated lipase of 568 U/L (normal: 13–60 U/L). Additionally, the white blood cell count, antinuclear antibody test, hemoglobin, ethanol level, triglyceride levels, electrolytes, and COVID‐19 PCR tests were all within normal ranges. Further imaging modalities such as chest radiography, abdominal ultrasound (US), and computed tomography of abdomen and pelvis (CTAP) with contrast were performed. The abdominal US showed a homogenous liver and no evidence of gallstones or gallbladder wall thickening, and no renal abnormalities. The CTAP with contrast showed minimal stranding adjacent to the mid duodenum and pancreatic head suggesting pancreatitis (Figure 1). Per Revised Atlanta classification, the patient made all three criteria so was diagnosed with AP. Further history revealed no recent trauma, no medication to prompt pancreatitis, steroid‐use, mumps, autoimmune, scorpion venom, or prior ERCP.

At the time of admission, the Harmless Acute Pancreatitis score was 0, and the Bedside Index of Severity in Acute Pancreatitis (BISAP) score was 1 given the patient's age. During the hospitalization, the patient received one liter intravenous (IV) bolus of 0.9% normal saline (NS) and continuous lactated ringers IV fluids at 250 ml/h for 10 hours. She was also given two doses of IV famotidine 20 mg with improvement and slowly being able to tolerate diet. Given her significant cannibal use history, the team strongly believe that it was not idiopathic but the diagnosis of cannabis‐induced pancreatitis (CIP) was made by diagnosis of exclusion. The patient was subsequently discharged after counseling regarding cessation of smoking tobacco and cannabis products.

## 3 DISCUSSION

Cannabis is increasingly becoming one of the most commonly used recreational substances and according to a survey by WHO, its use in the United States (US) and New Zealand are higher than other countries.^4^ The prevalence of cannabis use has doubled within a span of 10 years between 2001 and 2012.^5^ Changing regulations and ease of access have increased the prevalence of complications associated with cannabis use.

Cannabis induced pancreatitis is a rare cause of AP and the diagnosis is difficult because a clear diagnostic criteria is lacking. Cannabis was first reported as a possible cause of AP in 2004 and since then only 26 total cases have been reported. A systematic review by Barkin et al in 2017 describes a temporal relationship between the use of cannabis and recurrent pancreatitis in 15 of these 26 cases. Furthermore, the study showed that there were 13 reports of no further pancreatitis episodes after cannabis cessation.^6^ Although cannabis use has been associated with an increased incidence of AP, typically patients present earlier between 3 and 5 days after recent cannabis use.^7^ Manifestations of AP after prolonged use is rare but not entirely uncommon. In order to better understand the association between cannabis use and AP, a more detailed analysis of the underlying pathogenesis is important and required.

The pathogenesis of pancreatitis is proposed to be due to a “Three Hit mechanism” involving genetic, environmental, and an acute trigger leading to trypsinogen activation and, in turn, pancreatic injury. The genetic factors leading to pancreatitis have been studied extensively and several genes such as SPINK1, CFTR, HLA‐DRB*0401, and PRSS1 have been attributed with the increase in predisposition to develop pancreatitis.^8^, ^9^ These factors when combined with acute triggers such as pancreatic hyperstimulation, biliary disease, alcohol, medications, and recreational drugs lead to unregulated trypsin activity, rise in intra acinar calcium levels, zymogen activation, pancreatic autodigestion, and pancreatic inflammation.^10^, ^11^ However, the specific mechanisms involving the pathogenesis of CIP are poorly defined and understood. Currently, it is known that cannabis acts via two cannabinoid receptors CB I and CB II, which are both extensively prevalent in pancreatic tissue.^12^, ^13^ Dembinski et al demonstrated the physiological effects of CB I receptor with the help of anandamide, an endocannabinoid that caused significant increases in pancreatic amylase, lipase, ribonuclease, and IL‐1β secretions. It is thought that this interaction creates a physiological milieu similar to that which is noted in AP. Some other studies have also hypothesized that the activation of the CB I receptor leads to an impaired relaxation of the sphincter of oddi, which can further act as an acute trigger for CIP.^13^, ^14^

These cannabinoid receptors have also been noted to decrease gastric acid secretions, intestinal secretions, and delay gastric emptying.^13^ The relationship between cannabinoid receptors and the exocrine activity of the pancreas is well defined; however, its association with the endocrine functions remains unknown. Some suggest that the activation of CB1 receptors leads to a decrease in insulin secretion.^15^ Michalscki et al^16^ showed in a study that the activation of CB I and CB II receptors abolished the abdominal pain associated with pancreatitis. It is postulated that continued and prolonged use of cannabis will eventually lead to a compounding effect in the body triggering CIP in patients. As there can also be several other possible triggers to potentiate AP, establishing a strong causal link between cannabis use and AP is difficult. Further in‐depth molecular studies are needed to better define the roles of cannabinoid receptors and its physiological mechanisms regarding the pathophysiology involving CIP.

## 4 CONCLUSION

Cannabis is quickly becoming one of the most popular recreational drugs. Cannabis‐induced pancreatitis is uncommon, and identification is challenging due to a lack of precise diagnostic criteria. However, a thorough history and identifying risk factors becomes pivotal in preventing recurrences and can potentially curb the incidence of chronic pancreatitis and pancreatic cancer.

## CONFLICT OF INTEREST

None.

## AUTHOR CONTRIBUTIONS

DS, HG, SJ, and JR wrote the initial draft of the manuscript; GN, JL, DS and VJ edited the draft and reshaped it into this manuscript; all authors have approved the final version of the manuscript and agreed to be accountable for all aspect of the work in ensuring that question related to the accuracy or integrity of any part of the work are appropriately investigated and resolved.

## CONSENT

Written and verbal consent for this article has been obtained by the authors.

## Data Availability

Available upon request.
